# The term CAKUT has outlived its usefulness: the case for the prosecution

**DOI:** 10.1007/s00467-022-05576-4

**Published:** 2022-05-16

**Authors:** Adrian S. Woolf

**Affiliations:** 1 Division of Cell Matrix Biology and Regenerative Medicine, School of Biological Sciences, Faculty of Biology Medicine and Health, The University of Manchester, Michael Smith Building, Oxford Road, Manchester, M13 9PT UK; 2 Royal Manchester Children’s Hospital, Manchester Academic Health Science Centre, Manchester University NHS Foundation Trust, Manchester, UK

**Keywords:** Acronym, Bright’s disease, Bladder, Dysplasia, Gene, Kidney, Malformation, Semantics, Ureter

## Abstract

CAKUT stands for Congenital Anomalies of the Kidney and Urinary Tract, and the acronym first appeared in a review article published in 1998. Since then, CAKUT has become a familiar term encountered in the medical literature, especially in nephrology journals. I reason that the term CAKUT was conceived as not a simple description of various diseases, but more as shorthand for a bold conceptual package that linked the occurrence of diverse types of anatomical malformations with insights from genetic and developmental biology research. Moreover, the angiotensin II receptor type 2 was seen as a paradigmatic molecule in the pathobiology of CAKUT. I contend that the acronym, while appearing as an intellectually good idea at the time it was conceived, has outlived its usefulness. To reach these conclusions, I focus on the complex of research observations that led to the theory behind CAKUT, and then question whether these scientific foundations still stand firm. In addition, it is noted that not all clinicians have adopted the acronym, and I speculate why this is the case. I proceed to demonstrate that there is an incompatibility between the semantic meaning of CAKUT and the diseases for which the term was originally conceived. Instead, I suggest the acronym UTM, standing for Urinary Tract Malformation, is a simpler and less ambiguous one to use. Finally, I contend that the continued use of the acronym is a regressive step for the disciplines of nephrology and urology, taking us back two centuries when all kidney diseases were simply called Bright’s disease.

## Introduction

Through his dissection studies undertaken five centuries ago, Andreas Vesalius observed that the kidney was connected to the urinary bladder by the ureter, thus providing the anatomical basis for the idea that these organs acted as a single functional unit that makes and then excretes urine [1]. This conception of a harmonised upper and lower urinary tract will resonate with the day-to-day clinical experiences of pediatric nephrologists and also pediatric urologists who commonly care for children born with structural malformations of both the kidneys and the ureter, bladder, or urethra. Collectively, such congenital malformations have a prevalence of around 4 per 1000 births [2], and malformed kidneys are the most common cause of chronic kidney disease (CKD) stage 5 in young children [3]. Importantly, these disorders can also present with kidney failure in adulthood [4], in which context their congenital origin is not always immediately appreciated [4, 5]. It is currently recognised that a small subset of kidney and lower urinary tract malformations have defined monogenic causes [6, 7].

CAKUT stands for Congenital Anomalies of the Kidney and Urinary Tract, and the acronym was first used in the medical literature in 1998 [8]. Since then, CAKUT has become a familiar term encountered in the medical literature, especially in nephrology journals. I reason that the CAKUT acronym, while appearing a good idea at the time it was conceived, has outlived its usefulness. To reach this conclusion, this article first focuses on the collection of research observations that converged to lead the rationale for the term CAKUT. I then question whether these scientific foundations have stood the test of time. In addition, it is noted that not all clinicians have adopted the acronym, and I consider why this should be the case. I proceed to demonstrate that there is an incompatibility between the semantic meaning of CAKUT and the diseases for which the term was originally conceived. Instead, I suggest the acronym UTM, standing for Urinary Tract Malformation, which is simpler and less ambiguous. Finally, I contend that the continued use of the CAKUT acronym is a regressive step for the disciplines of nephrology and urology, taking us back two centuries when all kidney diseases were simply called Bright’s disease [9].

## How the acronym CAKUT was born

The acronym CAKUT first appeared in the medical literature in 1998 in a review article by Yerkes et al. [8]. The clinical data and laboratory experiments that underpinned the scientific bases for the acronym were not, however, detailed until the following year in an original research article from the same research group based at the Vanderbilt University Medical Center, Nashville, USA [10]. The research paper was entitled “Role of the angiotensin type 2 receptor gene in congenital anomalies of the kidney and urinary tract, CAKUT, of mice and men” and, according to Google Scholar, it had been cited over 300 times through the end of 2021. It is informative to analyse, and at times directly quote from, these two [8, 10] and related CAKUT publications [11, 12] from this research group.

Nishimura et al. [10] wrote “Prenatally diagnosed CAKUT are categorised as ureteropelvic junction stenosis or atresia (UPJ); multicystic dysplastic kidneys (MD), vesicoureteral reflux (VUR), megaureter (MU), ureteral duplication, and other less frequent ureteral anomalies; and bladder outlet obstruction”, and “There are a number of well-known but puzzling features in these abnormalities. Many are found more frequently in males”. In the same paper, they went on to write “As multiple CAKUT are often concurrently present, it is believed that these anomalies share a common pathogenic mechanism. These abnormalities often occur in a familial pattern”. The authors reasoned that their current research study explained these observations and that the new acronym CAKUT they then created encapsulated the spirit of these findings.

Nishimura et al. [10] reported that there existed a common polymorphism of *AGTR2* that codes for the angiotensin II receptor, type 2. The variant was shown to perturb the efficiency of *AGTR2* mRNA splicing. They further concluded that “…a remarkably strong association exists between the incidence of congenital anomalies of the kidney and urinary tract and the mutation”. These analyses were undertaken in Caucasian individuals from the USA and Germany comprising healthy controls and patients with UPJ. The same research paper explored urinary tracts of mice with null mutation of *AGTR2* and the authors wrote that these mice “…have phenotypes that remarkably resemble human CAKUT”. Around one-fifth of males and 5% of females had “grossly identifiable CAKUT”, mostly hydonephrosis, megaureter, or kidney dysplasia. Moreover, on cystometry, VUR was induced at lower hydrostatic pressures in null mutant mice compared with wild-type mice. Investigation of *AGTR2* null mutant embryonic mice showed an altered prevalence of apoptotic cell death around the stalk of the ureter [10]. Indeed, others had previously demonstrated that the fine control of programmed cell death is involved in sculpting developing nephrons [13].

A follow-up paper from the CAKUT research group [11] reported that in 60% of *AGTR2* null mutant mouse embryos there was an abnormal site of ureteric bud (UB) initiation from the pronephric, or Wolffian, duct. The UB is the epithelial tube that matures to form the urothelium in the stalk of the ureter as well as kidney collecting ducts [14]. It was envisaged that misplaced budding would result in either kidney dysplasia or VUR: the former because the UB tip would not completely engage with metanephric mesenchyme to induce nephrons, and the latter because the root of the UB would fail to form a non-refluxing junction with the bladder. The misplaced UB origin hypothesis fit in with historical clinical observations by Mackie and Stephens [15] who noted that, in individuals with duplex kidneys, kidney dysplasia correlated with aberrant insertion of the distal end of the ureter, itself presumably resulting from an aberrant origin of the UB.

In a review article summarising their breakthrough CAKUT research studies, Ichikawa and colleagues [12] wrote that “Ectopic budding of the ureter from the Wolffian duct is the first ontogenic misstep that leads to many—if not all—congenital anomalies of the kidney and urinary tract (CAKUT)”. This paper had been cited over 200 times through the end of 2021, as assessed by Google Scholar.

## Deconstructing the CAKUT concept

Therefore, the term CAKUT was conceived not as a simple description of various diseases, but more as shorthand for a bold conceptual package that linked the occurrence of diverse types of anatomical malformations with insights from genetic and developmental biology research. Moreover, the angiotensin II receptor 2 was seen as a paradigmatic molecule in the pathobiology of CAKUT. With the benefit of hindsight, let us now consider whether CAKUT’s underpinning scientific concepts still stand firm.

First, one can seriously question whether the *AGTR2* gene is indeed a key player in human malformations. A statistically significant association between the *AGTR2* A-G transition in intron 1 variant was found in an Italian cohort with VUR, kidney hypoplasia, MD, or UPJ [16]. In contrast, no such significant relationship was found in Japanese individuals with kidney hypoplasia, VUR, or UPJ [17]. Moreover, in a whole genome analysis of over 300 families with two or more siblings with VUR and/or reflux nephropathy, Cordell et al. found no significant association with single nucleotide polymorphisms in *AGTR2* [18]. Furthermore, a meta-analysis of studies of polymorphisms of the renin-angiotensin system in kidney diseases [19] did not support an association with the *AGTR2* variant studied by Nishimura et al. [10]. Finally, overtly pathogenic mutations of *AGTR2* (e.g. nonsense, frameshift, or deletion variants) that would parallel the null mutations of *AGTR2* mull mutant mice [10, 11] do not appear to have been reported in the medical literature on human urinary tract malformations.

Second, a key reason that the *AGTR2*/CAKUT link appeared attractive to Nishimura et al. [10] was because they had written “Many (types of CAKUT) are found more frequently in males” coupled with the fact that the *AGTR2* gene is on the X-chromosome; accordingly, males carrying an *AGTR2* variant on their sole X-chromosome would be predicted to have more severe disease than a female carrying one variant and one normal copy. Their simple statement about sex distribution in CAKUT [10], however, belies a more nuanced reality. It is correct that there is a strong male preponderance in kidney hypoplasia, at least in certain populations [17]. Congenital bladder outlet obstruction (BOO) was also conceived by Nishimura et al. [10] as being in the CAKUT spectrum. Indeed, posterior urethral valve (PUV) is a major cause of kidney failure in young males [20], and is a uniquely male disease. Mice with null mutations of *AGTR2* do not, however, have congenital BOO [10]. Thus, this inclusion of congenital BOO already sat uneasily within the original conception of CAKUT, yet this contradiction was barely emphasised by the research group. Finally, VUR was an important entity within the originally conceived CAKUT spectrum [10], yet familial VUR is in fact slightly more prevalent in females [21].

Third, how does the misplaced UB origin theory hold up as a cause for the various malformations in the CAKUT term? Certainly, there are other examples of developmental biology studies where mutations of genes other than *AGTR2* have linked kidney malformations with an aberrant bud origin. An example is provided by *forkhead box C1* (*FOXC1*) mutant mice that are born with duplex ureters and malformed kidneys [22]. On the other hand, other evidence indicates that the most severe CAKUT phenotype, namely kidney agenesis (i.e. an absent kidney usually accompanied by an absent ureter) can be caused not by an ectopic origin of the UB, but by a lack of UB emergence from the Wolffian duct with subsequent death of nephron precursors in the metanephric mesenchyme [23, 24]. For example, Fraser syndrome commonly features kidney agenesis [25] and is caused by mutations of Fraser extracellular matrix complex subunit 1 (*FRAS1)* or related genes such as *FREM2* [25, 26]. These code for proteins that, in health, coat the surface of the emerging UB and which enhance growth factor signalling between metanephric mesenchyme and the bud [23, 24]. Another example is provided by kidney dysplasia caused by mutations of *hepatocyte nuclear factor 1B* (*HNF1B*); this is currently among the most common genetically defined causes of a CAKUT [27]. Modelling the kidney malformation in mutant mice [28] or with human pluripotent stem cell–derived organoid technology [29] each suggests that the fundamental pathobiology is caused by aberrant tubule differentiation within the kidney itself rather than being initiated by a misplaced UB origin.

Finally, emerging work suggests that ectopic UBs do not explain congenital BOO. While possible genetic bases of PUV remain to be defined [30], it has been postulated that an aberrant insertion of the distal end of the Wolffian duct into the proximal urethra is the underlying pathobiology generating PUV [31]. On the other hand, duplicated ureters that would be consistent with UB ectopia are not typically associated with PUV. Moreover, congenital BOO associated with, for example, bladder dyssynergia or detrusor hypocontactility can be caused by mutations of genes involved in bladder innervation [32] or detrusor smooth muscle maturation [33], i.e. nothing to do with the biology of the UB.

## The variability of CAKUT phenotypes within single families

It has been noted that when CAKUT runs in families, the particular type of malformation can vary between affected individuals [34]. For example, Kerecuk et al. [5] described striking variable expressivity in a three-generation family with autosomal dominant disease. Some individuals had severe kidney dysplasia leading to CKD stage 5, while others had mild kidney hypoplasia with minimal impact on kidney excretory function [5]. Even more extreme examples have been described where carrying a gene variant was only associated with a kidney malformation in a subset of individuals [35].

What might account for this variability is under investigation. One explanation could be that a more severe phenotype requires that the affected person carries variants in more than one gene, as described for *Paired box 2* and *Sine oculis 1* [36]. Another explanation involves exposure of the developing kidney to harmful environmental influences that vary between pregnancies. Indeed, human clinical studies as well as animal experiments suggest that a decrease in maternal ingestion of major dietary constituents such as protein [37, 38], or maternal diabetes mellitus [39–41], or altered vitamin intake in pregnancy [40, 42] can each compromise kidney development.

Thus, variable expressivity may have complex genetic and/or environmental origins. It is here acknowledged that this concept may be more easily explained to families by counselling them that they have a propensity to a disorder called ‘CAKUT’, a term that includes different phenotypes. On the other hand, while it is correct that, for example, kidney hypoplasia and kidney dysplasia can occur in one kindred [5], the ‘CAKUT concept’ could appear less useful as a genetic counselling tool in other circumstances. For example, a family with congenital BOO caused by PUV would be very unlikely to be at increased risk for kidney agenesis, or vice versa, because these are diseases with very different mechanisms.

## A question of the meaning of the words that make up CAKUT

It has been commented that the medical community is attracted to using acronyms [43] and that “Within the health sciences, researchers’ use of acronyms holds a long tradition, with the likely intention of branding their work into the minds of fellow researchers, clinicians, editors, or lay people”. [44]. The idea behind certain acronyms is simple; for example, HNF1B is the acronym for the molecule called hepatocyte nuclear factor 1B [27]. While the meaning of such an example is clear, and its usefulness as standard shorthand would not be disputed, these attributes are much less clear for CAKUT. Indeed, as discussed above, the term CAKUT was coined to cover a translational research concept, linking mice and humans, as much as to refer to a disease, or group of diseases.

Clinicians and scientists are professionals who typically demand precise language in their daily work, but does the term CAKUT serve them well in this manner? What, exactly, does the acronym CAKUT stand for, and do the individual words even make semantic sense? Let us start by breaking down the individual words and phrases using established dictionaries [45, 46]. The definition of the adjective ‘congenital’ is “present from birth” and ‘anomaly’ is a noun meaning “something that deviates from what is normal”. Also useful is the World Health Organization definition [47] that “Congenital anomalies can be defined as structural or functional anomalies that occur during intrauterine life”. Here, one can immediately see an ambiguity in the term CAKUT. Yerkes et al. [8] and Nishimura et al. [10] were clearly using the term to describe gross structural anomalies, or malformations, but not functional anomalies that occurred in the face of intact gross anatomy. The latter would logically include important disorders such as congenital nephrotic syndrome [48], functional kidney tubule diseases presenting before birth [49], and prenatal kidney vein thrombosis [50]. Moreover, even with respect to gross structural malformations, Yerkes et al. [8] and Nishimura et al. [10] did not include the polycystic kidney diseases presenting before birth with enlarged kidneys [51, 52].

Moving along in breaking down the meaning of individual words that comprise CAKUT, the word ‘kidney’ should pose little challenge to readers of this journal. The dictionary definition is “one of a pair of vertebrate organs situated in the body cavity near the spinal column that excrete waste products of metabolism”. In contrast, the definition of the phrase ‘urinary tract’ may be less familiar and the dictionary definition is “The tract through which urine passes and which consists of the renal tubules and renal pelvis of the kidney, the ureters, the bladder, and the urethra”. Even more explicitly, according to the National Institute of Diabetes and Digestive and Kidney Diseases [53], the ‘urinary tract’ comprises: “The organs of the body that produce, store, and discharge urine. These organs include the kidneys, ureters, bladder, and urethra”. In other words, the word “kidneys” within CAKUT is redundant.

Putting this all together, a more accurate acronym to describe the collection of CAKUT disorders as originally conceived by Yerkes et al. [8] and Nishimura et al. [10] would simply be ‘urinary tract malformations’ or ‘UTMs’. Here, I am using the word ‘malformation’ in its dictionary definition of “irregular or abnormal structural development”. Within this term, I would semantically include congenital presentations of polycystic kidney disease in addition to kidney dysplasia, kidney hypoplasia, UPJ, VUR, and congenital BOO.

## Who uses the term CAKUT?

As assessed by a PubMed search on 23 December 2021, there had been 545 papers published since 1998 that had “CAKUT” in the title of the paper and/or the abstract. Since its inception in 1998, the use of the acronym appears to have reached a plateau, with between 81 and 89 publications per year recorded in the last three years of the search. Classifying journals by topic, CAKUT was most popular in kidney journals, with a total of 26 papers in *Pediatric Nephrology*, 22 in *Kidney International*, and 15 in *Journal of the American Society of Nephrology*. The second most popular speciality appeared to be genetics, for example with eight publications in the *American Journal of Medical Genetics*, seven in the *American Journal of Human Genetics*, and six in *Human Molecular Genetics*. In fact, considering all the articles, the word “gene” plus “CAKUT” appeared in the title or abstract in 235 (43%) of the total 545. Of note, in this CAKUT/gene subset, around 20% of the publications came from just three prolific research groups. This is consistent with observations by Pottegård and colleagues [44] that medical acronyms are often adopted by authors to “brand”, and thus help own and publicise, their work.

A very different perspective is apparent when looking at urology journals, with few articles that invoke “CAKUT”. Strikingly, the *Journal of Pediatric Urology*, a publication with many articles about urinary tract malformations, only had only five articles with “CAKUT” in the title or abstract. Furthermore, the *Journal of Urology*, a publication with a subsection devoted to pediatric urology, had only five articles featuring “CAKUT”. Thus, urology journals rarely use the acronym, most probably because it alone transmits no useful anatomical information and surgeons need to know the precise anatomical diagnosis such as PUV, UPJ, or VUR. It is also the experience of this author, having worked in several UK nephrology departments since the inception of the CAKUT term, that the acronym is not generally used in everyday clinical practice, for example as written in clinic notes or referral letters. Instead, specific anatomical diagnoses are used in clinic notes, in-patient summaries, and operation notes. Summarising these observations, “CAKUT” is typically used in the context of a research article with the focus on possible genetic bases, rather than the precise anatomy, of urinary tract malformations.

## A retrograde step for nephrologists

As reviewed in great detail by Cameron [9], two hundred years ago, Richard Bright became an influential figure in cutting-edge medicine. Bright linked observations of peripheral oedema and albuminuria in life with diseased kidneys on the autopsy table. Thus ‘Bright’s disease’ was born and this term subsequently dominated the emerging field of nephrology for over a century. Much like the ectopic UB theory to explain the genesis of all types of CAKUT, Bright hypothesised that his patients with kidney disease shared a common pathobiology that involved an early phase involving increased blood flow. In retrospect, we now believe that Bright’s original cases, and others for which the term was subsequently used as a diagnosis, may have had any one of a range of specific kidney diseases including the various types of glomerulonephritis, diabetic nephropathy, amyloidosis, as well as Alport syndrome. Indeed, modern nephrologists would never use the term ‘Bright’s disease’ because they pride themselves on making any one out of several tens of specific kidney diagnoses, aided by information from, for example, kidney biopsies and genetic analyses.

With the above in mind, we need to ask why a modern nephrologist would want to use the term ‘CAKUT’ when this contains such a wide range of structural malformations and also given that, as discussed earlier, there is no one developmental biology mechanism or genetic cause that links all these malformations. Another way of considering this is to ask whether modern nephrologists would adopt a new acronym covering diseases acquired after birth to complement, or as a mirror image, the term CAKUT. Let us call such a term “AAKUT”, or Acquired Anomalies of the Kidney and Urinary Tract. AAKUT would encompass all structural anomalies of the tract including hydronephrosis secondary to a ureteric stone or prostatic enlargement, as well as the gross distortions of kidney shape produced by autosomal dominant kidney disease or recurrent scarring episodes of pyelonephritis. This author believes that AAKUT would never be adopted by the current clinical and research communities and would be seen as a regressive step back to the era of ‘Bright’s disease’. If one agrees with that line of thought, why would one ever want to use CAKUT?

## Summary

Table 1 summarises some of the main objections we have made to using the acronym CAKUT. We contend that it is time to seriously question whether the term should be perpetuated in clinical practice or in research because its meaning is ambiguous, and the term lacks anatomical precision. Moreover, there is no compelling scientific reason to believe that the group of diseases encompassed by CAKUT has a single disease mechanism, as originally believed. If one wanted a simpler term to describe the anatomic anomalies, then use ‘Urinary Tract Malformations’. Don’t, however, use the UTM acronym as a substitute for specifying the exact malformation, nor as a pseudo-scientific jargon term to imply a common developmental pathobiology.

**Table 1:** Reasons not to use the acronym called CAKUT

1. The collection of scientific bases (including a misplaced origin of the ureter bud) underpinning the CAKUT concept are too simplistic to explain the range of kidney, ureter, and bladder structural malformations encompassed by the term
2. The linguistic meaning of the words that make up CAKUT show repetition (i.e. ‘kidney’ is redundant because it is part of the ‘urinary tract’), and the word ‘anomaly’ would logically include functional anomalies (e.g. congenital nephrotic syndrome) in addition to the gross structural malformations meant by the creators of the term CAKUT
3. Even if we confine using the term CAKUT to structural malformations, the term transmits no precise anatomical information
4. Clinicians and scientists would never accept using the mirror image term “AAKUT” (Acquired Anomalies of the Kidney and Urinary Tract) as an umbrella term for non-congenital disease, so why would they use CAKUT?
5. Lumping together all congenital diseases of the kidney and lower urinary tract is akin to using the out of date term ‘Bright’s disease’, and is thus a retrograde step for our speciality
